# The Labors of State Boards of Medical Examiners and Licensers and the State and Other Boards of Health

**Published:** 1897-12

**Authors:** 


THE labors of the State Boards of Medical Examiners and Licensers, and the State and other Boards of Health are made manifest:

By the improvement in the material from which medical men are formed. By the better preliminary education of medical students.

By the higher technical education of doctors.

By the binding together of the best medical colleges in a league, the sole object of which is to elevate the standard of medical education.

By the enactment of laws which admit only properly equipped men to enter the practice of medicine.

By the rapid disappearance of the pretender and ignorant quack.

By the presence in every village or hamlet of a medical man whose character and attainments entitle him to rank with the best of his community; whose professional acumen and honesty fit him to discharge the duties of his high calling with confidence, skill and success, and whose trained faculties constitute him the guardian of the public health.—*Med. and Surg. Reporter.*

Fifty-one metals are now known to exist; four hundred years ago only seven were known.

